# Competing Risk Analyses of Medullary Carcinoma of Breast in Comparison to Infiltrating Ductal Carcinoma

**DOI:** 10.1038/s41598-019-57168-2

**Published:** 2020-01-17

**Authors:** Dongjun Dai, Rongkai Shi, Zhuo Wang, Yiming Zhong, Vivian Y. Shin, Hongchuan Jin, Xian Wang

**Affiliations:** 1 Department of Medical Oncology, Sir Run Run Shaw Hospital, Medical School of Zhejiang University, Hangzhou, China; 2 Laboratory of Cancer Biology, Key Lab of Biotherapy, Sir Run Run Shaw Hospital, Medical School of Zhejiang University, Hangzhou, China; 3 Department of Surgery, Queen Mary Hospital, The University of Hong Kong, Pokfulam, Hong Kong SAR China

**Keywords:** Breast cancer, Risk factors

## Abstract

The aim of current study was to use competing risk model to assess whether medullary carcinoma of the breast (MCB) has a better prognosis than invasive ductal carcinomas of breast cancer (IDC), and to build a competing risk nomogram for predicting the risk of death of MCB. We involved 3,580 MCB patients and 319,566 IDC patients from Surveillance, Epidemiology, and End Results (SEER) database. IDC was found to have a worse BCSS than MCB (Hazard ratio (HR) > 1, p < 0.001). The 5-year cumulative incidences of death (CID) was higher in IDC than MCB (p < 0.001). Larger tumor size, increasing number of positive lymph nodes and unmarried status were found to worsen the BCSS of MCB (HR > 1, p < 0.001). We found no association between ER, PR, radiotherapy or chemotherapy and MCB prognosis (p > 0.05). After a penalized variable selection process, the SH model-based nomogram showed moderate accuracy of prediction by internal validation of discrimination and calibration with 1,000 bootstraps. In summary, MCB patients had a better prognosis than IDC patients. Interestingly, unmarried status in addition to expected risk factors such as larger tumor size and increasing number of positive lymph nodes were found to worsen the BCSS of MCB. We also established a competing risk nomogram as an easy-to-use tool for prognostic estimation of MCB patients.

## Introduction

Medullary carcinoma of the breast (MCB) is a rare histology type of invasive breast cancer, accounting for 3–5% of all breast cancers. The histopathologic features of MCB include lymphoplasmacytic infiltration, noninvasive microscopic circumscription, syncytial growth pattern >75%, and grade 2 or 3 nuclei^1^. Immunohistochemical results showed that MCB presented with more absence of estrogen (ER), progesterone (PR), and human epidermal growth factor receptor 2 (HER2)^2^. Compared with invasive ductal carcinomas of breast cancer (IDC), MCB also exhibited with more BRCA1/2 mutations, greater T stage and tumor size^3^.

Despite the aggressive features, the prognosis of MCB was conflicting. Studies found MCB showed a better survival than IDC^1,3–6^, while other researches showed MCB (IDC) had a similar outcomes with IDC^7–9^. However, since MCB is a rare type of breast cancer, most of these studies were involved with limited samples. Surveillance, Epidemiology, and End Results (SEER) database of the National Cancer Institute is a national collaboration program of Unite State, covering nearly 26% American population’s cancer incidence and survival data, which could provide relatively large sample size for survival study. There were two SEER-based studies associated with MCB prognosis^7,10^. One study^7^ only focused on short-term survival status. The inadequate follow-up time might lead to skewed results. The other study^10^ evaluated the prognostic factors of long-term MCB overall survival (OS) by Cox proportional hazards model. However, OS might unable to accurately describe long-term survival of disease such MCB, as the deaths could be caused by other competing risk events. In this case, the cumulative incidence function (CIF) model^11^ and Fine-Gray regression for proportional hazards modeling of the subdistribution hazard (SH) model^12^ should be used.

Duo to the limitation of previous studies, we designed this competing risk model-based study, which involved with SEER data, to evaluate whether MCB has a better prognosis than with IDC in early resectable breast cancer patients, and to evaluate the long-term prognostic value of different clinical factors in MCB. Furthermore, since there was no nomogram drawing for the prognosis of MCB patients, the current study also constructed a competing risk nomogram for MCB patients.

## Results

### Cohort selection

After selection, we involved 3,580 MCB patients and 319,566 IDC patients (Table 1). There were significant differences between MCB and IDC among age, race, location, grade, tumor size, tumor stage, number of positive regional nodes, ER or PR status, and chemotherapy experience (*p* < 0.001, Table 1). Compared with IDC, the MCB patients presented with younger age (age < 50, 47.4% vs. 28.8%), more African Americans (21% vs. 9.3%), higher grade (grade III and IV, 63.8% vs. 39.9%), larger tumor size (>2 cm, 51.2% vs. 34.9%), higher proportion-Adjusted AJCC 6th Stage II (54.8% vs. 36.8%), less positive regional nodes (no positive node, 71.9% vs. 65.3%), more ER negative (78.5% vs. 24.8%) and PR negative (81.7% vs. 34.4%) rate, and more experience of chemotherapy (61% vs. 46.2%). The median follow-time were 143 months (interquartile range [IQR], 94 to 204 months) for MCB and 107 months (IQR, 73 to 155 months) for IDC. The median age of diagnosis was 50 years (IQR, 43 to 60 years) for MCB and 57 years (IQR, 48 to 67 years) for IDC. The rate of breast cancer specific mortality (BCSM) and other causes of death were 11.06% and 14.19% for MCB and 13.14% and 15.8% for IDC, respectively. The proportion of deaths due to cancer and other causes in each variable was listed in Supplemental Table 1, from which we observed the deaths caused by other reasons increased rapidly as the age raised.

**Table 1:** The characteristic of each involved variable in MCB and IDC.

Characteristics	MCB		IDC		p value
	No. of patients	%	No. of patients	%	
**Age**					**<0**.**001**
20–29	66	1.8	2048	0.6	
30–39	523	14.6	20875	6.5	
40–49	1108	30.9	69132	21.6	
50–59	954	26.6	87673	27.4	
60–69	629	17.6	79286	24.8	
70–79	300	8.4	60552	18.9	
**Race**					**<0**.**001**
Caucasian	2554	71.3	262815	82.2	
African American	751	21	29576	9.3	
American Indian/Alaska Native	30	0.8	1585	0.5	
Asian or Pacific Islander	245	6.8	25590	8	
**Laterality**					0.339
Right - origin of primary	1795	50.1	157619	49.3	
Left - origin of primary	1785	49.9	161947	50.7	
**Location**					**<0**.**001**
Nipple	6	0.2	1443	0.5	
Central portion of breast	109	3	17224	5.4	
Upper-inner quadrant	408	11.4	36352	11.4	
Lower-inner quadrant	220	6.1	18796	5.9	
Upper-outer quadrant	1459	40.8	118097	37	
Lower-outer quadrant	281	7.8	22607	7.1	
Axillary tail	60	1.7	2284	0.7	
Overlapping lesion	711	19.9	66669	20.9	
Breast, NOS	326	9.1	36094	11.3	
**Grade**					**<0**.**001**
Well differentiated; Grade I	24	0.7	52366	16.4	
Moderately differentiated; Grade II	182	5.1	125171	39.2	
Poorly differentiated; Grade III		2058	57.5	122271	38.3
Undifferentiated; anaplastic; Grade IV	227	6.3	5267	1.6	
Unknown	1089	30.4	14491	4.5	
**Tumor size**					**<0**.**001**
**<** = 1 cm	316	8.8	78845	24.7	
**<** = 2 cm	1431	40	129280	40.5	
**<** = 3 cm	1145	32	63200	19.8	
**<** = 4 cm	414	11.6	23283	7.3	
**<** = 5 cm	151	4.2	10539	3.3	
> 5 cm	123	3.4	14419	4.5	
**Tumor stage**					**<0**.**001**
I	1332	37.2	158594	49.6	
II	1961	54.8	117460	36.8	
III	275	7.7	39923	12.5	
IV	12	0.3	3589	1.1	
**Regional nodes positive**					**<0**.**001**
> = 10	55	1.5	11196	3.5	
0	2574	71.9	208705	65.3	
1–3	782	21.8	74811	23.4	
4–9	169	4.7	24854	7.8	
**ER status**					**<0**.**001**
Negative	2810	78.5	79237	24.8	
Positive	727	20.3	238967	74.8	
Borderline	43	1.2	1362	0.4	
**PR status**					**<0**.**001**
Negative	2925	81.7	109903	34.4	
Positive	621	17.3	207188	64.8	
Borderline	34	0.9	2475	0.8	
**Marital status**					0.365
Married	2276	63.6	200768	62.8	
Unmarried	1304	36.4	118798	37.2	
**Radiotherapy**					0.975
No	1591	44.4	141889	44.4	
Yes	1989	55.6	177677	55.6	
**Chemotherapy**					**<0**.**001**
No	1397	39	172053	53.8	
Yes	2183	61	147513	46.2	

MCB, medullary carcinoma of the breast; IDC, invasive ductal carcinoma of breast; p value was calculated by Chi-Squared tests; Significant results were bolded.

### MCB showed better outcomes than IDC

**Figure 1:**
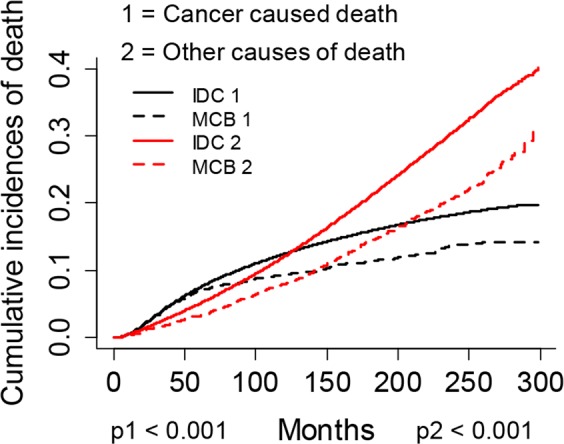
Cumulative incidence plots of competing risk of breast cancer specific deaths and other causes of deaths for cohort of MCB and IDC.

As shown in Table 2, We found IDC had a higher 5-years cumulative incidences of death (CID) of both breast cancer specific survival (BCSS) (*p* < 0.001) and other causes of deaths (*p* < 0.001) than the MCB (Fig. 1). The multivariate SH model found IDC had worse BCSS than MCB (Hazard ratio (HR) > 1). Furthermore, since statistical matching methods could lower the differences between groups on confounding variables and made cancer observation studies in somehow be considered as a quasi-experimental study, we performed a coarsened exact matching (CEM) between MCB and IDC by matching all the included variables. The standardized difference in means of all variables between MCB and IDC was eliminated and the histograms of IDC and MCB looked much more similar after CEM (see Supplementary Fig. S1-2 online), indicating our matching was successful. Matched 2,307 MCB patients and 24,398 IDC patients were further analyzed by CIF test and multivariate SH model. The new results remained as pre-matched analyses (Table 2). These multi-face results showed IDC had worse BCSS than MCB in resectable breast cancer patients.

**Table 2:** The results of CIF test and multivariate SH model between MCB and IDC prognosis before and after CEM.

Status	Type	CIF test				Multivariate SH model			
		5-year CID of cancer	p value	5-year CID of other causes	p value	HR (95%CI)	p value	se (coef)	Z value
Before matching	MCB	0.069	**<0**.**001**	0.032	**<0**.**001**	**Reference**	**<0**.**001**	0.09	6.19
	IDC	0.074		0.05		1.79 (1.49, 2.15)			
After matching	MCB	0.054	**<0**.**001**	0.028	0.061	**Reference**	**<0**.**001**	0.08	7.99
	IDC	0.07		0.035		1.84 (1.59, 2.14)			

CIF, cumulative incidence function; CID, cumulative incidences of death; MCB, medullary carcinoma of the breast; IDC, invasive ductal carcinoma of breast; HR, hazard ratio; 95%CI, 95% confidence index; se (coef), standard error of the regression coefficient; Significant results were bolded.

### Multivariate SH Analysis of BCSS in IDC and MCB

The prognostic value of each involved variable for IDC and MCB BCSS was listed in Table 3. Larger tumor size, a greater number of positive regional lymph nodes and unmarried status were found to be risk factors for BCSS of both IDC and MCB (HR > 1, *P* < 0.05). Meanwhile there were prognostic discrepancies among other variables between IDC and MCB. We found the location of nipple was a risk factor for MCB (Upper-outer quadrant vs. nipple, HR < 1, *p* = 0.043) but was a protective factor for IDC (Upper-inner quadrant or lower-inner quadrant vs. nipple, HR > 1, *p* < 0.05). Higher grade showed a worse BCSS of IDC (HR > 1, *p* < 0.001). However, higher grade showed no association with MCB BCSS (III/II vs. I, p > 0.05) or even had a better BCSS (IV vs. I, HR < 1, *p* = 0.013). In addition, we found age, race, Breast-Adjusted AJCC 6th Stage, ER and PR status and treatments of radiotherapy or chemotherapy were significantly correlated with IDC (*p* < 0.05) but not MCB (*p* > 0.05). Specifically, we found older age (40–69 vs. 20–29) was a protective factor (HR < 1, *p* < 0.05) of IDC. Compared with Caucasians, the African American and American Indian/Alaska Native had worse outcomes (IDC, HR > 1, *p* < 0.05). The Asian or Pacific Islander showed a better BCSS than Caucasians (IDC, HR < 1, *p* < 0.001). Higher stage increased the BCSS of IDC (HR > 1, *p* < 0.001). Positive ER or PR status and radiotherapy or chemotherapy lower the BCSS of IDC (HR < 1, *p* < 0.05). There was no association between laterality and BCSS in both IDC and MCB (*p > *0.05).

**Table 3:** The results of multivariate SH model of each variable for MCB and IDC.

Variable	Multivariate SH model							
	MCB				IDC			
	HR (95%CI)	p value	se (coef)	Z value	HR (95%CI)	p value	se (coef)	Z value
**Age**								
20–29	**Reference**				**Reference**			
30–39	1.15 (0.53–2.50)	0.72	0.40	0.35	0.92 (0.77–1.09)	0.33	0.09	−0.98
40–49	1.03 (0.48–2.20)	0.94	0.39	0.08	0.74 (0.62–0.87)	**<0**.**001**	0.09	−3.51
50–59	1.18 (0.55–2.53)	0.66	0.39	0.44	0.74 (0.62–0.87)	**<0**.**001**	0.09	−3.53
60–69	1.30 (0.60–2.82)	0.51	0.39	0.67	0.78 (0.65–0.92)	**0**.**004**	0.09	−2.92
70–79	1.22 (0.54–2.73)	0.63	0.41	0.48	0.88 (0.74–1.04)	0.14	0.09	−1.49
**Race**								
White	**Reference**				**Reference**			
Black	1.27 (0.99–1.62)	0.061	0.13	1.87	1.30 (1.23–1.37)	**<0**.**001**	0.03	9.52
American Indian/Alaska Native	1.34 (0.56–3.24)	0.51	0.45	0.65	1.28 (1.02–1.61)	**0**.**037**	0.12	2.09
Asian or Pacific Islander	0.95 (0.61–1.48)	0.82	0.23	−0.22	0.82 (0.77–0.88)	**<0**.**001**	0.04	−5.43
**Laterality**								
Right - origin of primary	**Reference**				**Reference**			
Left - origin of primary	0.95 (0.78–1.17)	0.64	0.10	−0.46	1.03 (1.00–1.07)	0.073	0.02	1.79
**Location**								
Nipple	**Reference**				**Reference**			
Central portion	0.36 (0.09–1.56)	0.17	0.74	−1.36	1.13 (0.88–1.46)	0.33	0.13	0.97
Upper-inner quadrant	0.34 (0.08–1.36)	0.13	0.71	−1.53	1.31 (1.03–1.68)	**0**.**031**	0.13	2.16
Lower-inner quadrant	0.30 (0.07–1.25)	0.097	0.73	−1.66	1.30 (1.01–1.67)	**0**.**044**	0.13	2.01
Upper-outer quadrant	0.24 (0.06–0.96)	**0**.**043**	0.70	−2.02	1.06 (0.83–1.35)	0.66	0.12	0.44
Lower-outer quadrant	0.31 (0.08–1.28)	0.11	0.72	−1.62	1.17 (0.91–1.50)	0.23	0.13	1.20
Axillary tail	0.20 (0.04–1.06)	0.059	0.85	−1.89	1.11 (0.81–1.53)	0.52	0.16	0.65
Overlapping lesion	0.30 (0.08–1.20)	0.088	0.71	−1.71	1.18 (0.92–1.50)	0.19	0.13	1.31
Breast, not otherwise specified	0.31 (0.08–1.25)	0.099	0.71	−1.65	1.26 (0.99–1.61)	0.066	0.13	1.84
**Grade**								
Well differentiated; Grade I	**Reference**				**Reference**			
Moderately differentiated; Grade II	0.62 (0.21–1.80)	0.38	0.55	−0.89	1.87 (1.72–2.04)	**<0**.**001**	0.04	14.23
Poorly differentiated; Grade III	0.40 (0.15–1.11)	0.079	0.52	−1.75	2.59 (2.38–2.83)	**<0**.**001**	0.04	21.36
Undifferentiated; anaplastic; Grade IV	0.24 (0.08–0.75)	**0**.**013**	0.57	−2.47	2.80 (2.44–3.21)	**<0**.**001**	0.07	14.69
Unknown	0.39 (0.14–1.08)	0.071	0.52	−1.81	2.47 (2.21–2.76)	**<0**.**001**	0.06	16.12
**Tumor size**								
**<** = 1 cm	**Reference**				**Reference**			
**<** = 2 cm	1.42 (0.87–2.33)	0.16	0.25	1.41	1.75 (1.63–1.88)	**<0**.**001**	0.04	15.73
**<** = 3 cm	1.65 (0.95–2.86)	0.077	0.28	1.77	2.05 (1.89–2.22)	**<0**.**001**	0.04	17.12
**<** = 4 cm	2.12 (1.17–3.84)	**0**.**013**	0.30	2.47	(	**<0**.**001**	0.05	19.82
**<** = 5 cm	2.95 (1.51–5.78)	**0**.**002**	0.34	3.16	2.61 (2.35–2.89)	<**0**.**001**	0.05	18.43
> 5 cm	4.69 (2.43–9.08)	**<0**.**001**	0.34	4.60	2.55 (2.31–2.81)	**<0**.**001**	0.05	18.77
**Tumor stage**								
I	**Reference**				**Reference**			
II	1.12 (0.73–1.71)	0.6	0.22	0.52	1.39 (1.29–1.50)	**<0**.**001**	0.04	8.66
III	0.81 (0.36–1.81)	0.61	0.41	−0.51	2.25 (2.00–2.52)	**<0**.**001**	0.06	13.83
IV	2.79 (0.64–12.10)	0.17	0.75	1.37	6.53 (5.74–7.44)	**<0**.**001**	0.07	28.32
**Regional nodes positive**								
> = 10	**Reference**				**Reference**			
0	0.12 (0.05–0.26)	**<0**.**001**	0.41	−5.28	0.35 (0.31–0.38)	**<0**.**001**	0.05	−20.11
1–3	0.24 (0.11–0.51)	**<0**.**001**	0.39	−3.71	0.56 (0.51–0.61)	**<0**.**001**	0.05	−12.47
4–9	0.77 (0.43–1.37)	0.37	0.30	−0.89	0.68 (0.64–0.73)	**<0**.**001**	0.03	−11.08
**ER status**								
Negative	**Reference**				**Reference**			
Positive	0.96 (0.70–1.34)	0.83	0.17	−0.22	0.82 (0.78–0.87)	**<0**.**001**	0.03	−6.87
Borderline	0.56 (0.15–2.03)	0.38	0.66	−0.89	1.14 (0.92–1.42)	0.24	0.11	1.18
**PR status**								
Negative	**Reference**				**Reference**			
Positive	0.99 (0.71–1.40)	0.97	0.17	−0.03	0.82 (0.78–0.87)	**<0**.**001**	0.03	−7.49
Borderline	0.78 (0.17–3.64)	0.75	0.79	−0.32	0.93 (0.77–1.13)	0.48	0.10	−0.71
**Marital status**								
Married	**Reference**				**Reference**			
Unmarried	1.33 (1.07–1.65)	**0**.**01**	0.11	2.59	1.11 (1.07–1.15)	**<0**.**001**	0.02	5.66
**Radiotherapy**								
No	**Reference**				**Reference**			
Yes	1.15 (0.92–1.43)	0.21	0.11	1.26	0.91 (0.88–0.95)	**<0**.**001**	0.02	−4.89
**Chemotherapy**								
No	**Reference**				**Reference**			
Yes	0.84 (0.67–1.07)	0.16	0.12	−1.41	0.94 (0.90–0.99)	**0**.**01**	0.02	−2.57

SH model, Fine-Gray regression for proportional hazards modeling of the subdistribution hazard model; MCB, medullary carcinoma of the breast; IDC, invasive ductal carcinoma of breast; HR, hazard ratio; 95%CI, 95% confidence index; se (coef), standard error of the regression coefficient; ER, estrogen; PR, progesterone; Significant results were bolded.

### Nomogram development and validation

**Figure 2:**
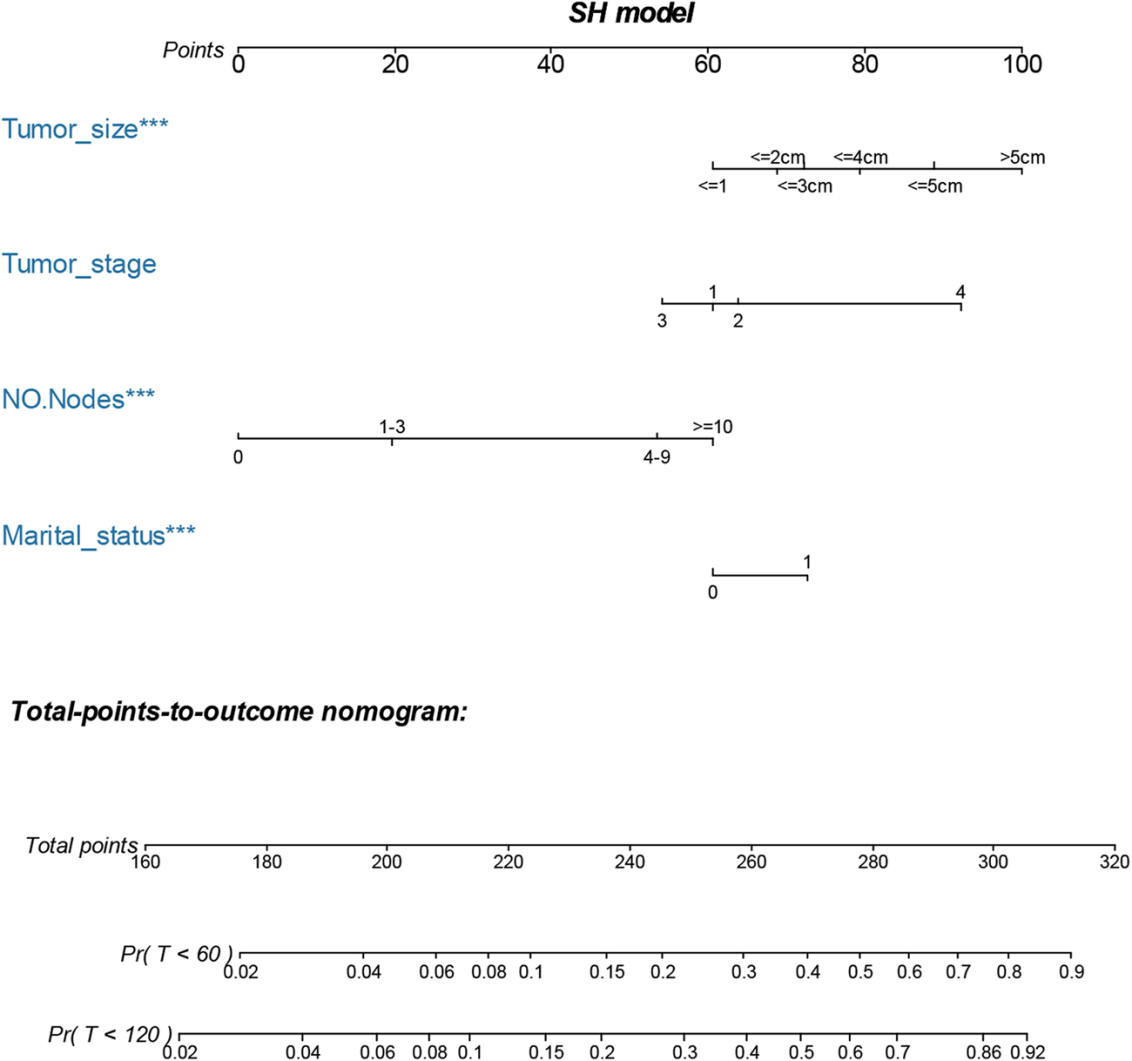
SH model-based nomogram for predicting 5- and 10-year risk of death of MCB patients. The nomogram is used by summing the points identified on the top scale for each independent variable and drawing a vertical line from the total points scale to the 5- and 10-year (60 and 120 months) risk of death. The total points projected to the bottom scale indicate the % probability of the 5- and 10-year risk of death. Tumor stage, 1 = Stage I, 2 = Stage II, 3 = Stage III and 4 = Stage IV; NO. Nodes, the number of positive regional lymph nodes; Marital status: 0 = married; 1 = widowed or single (never married or having a domestic partner) or divorced or separated.

**Figure 3:**
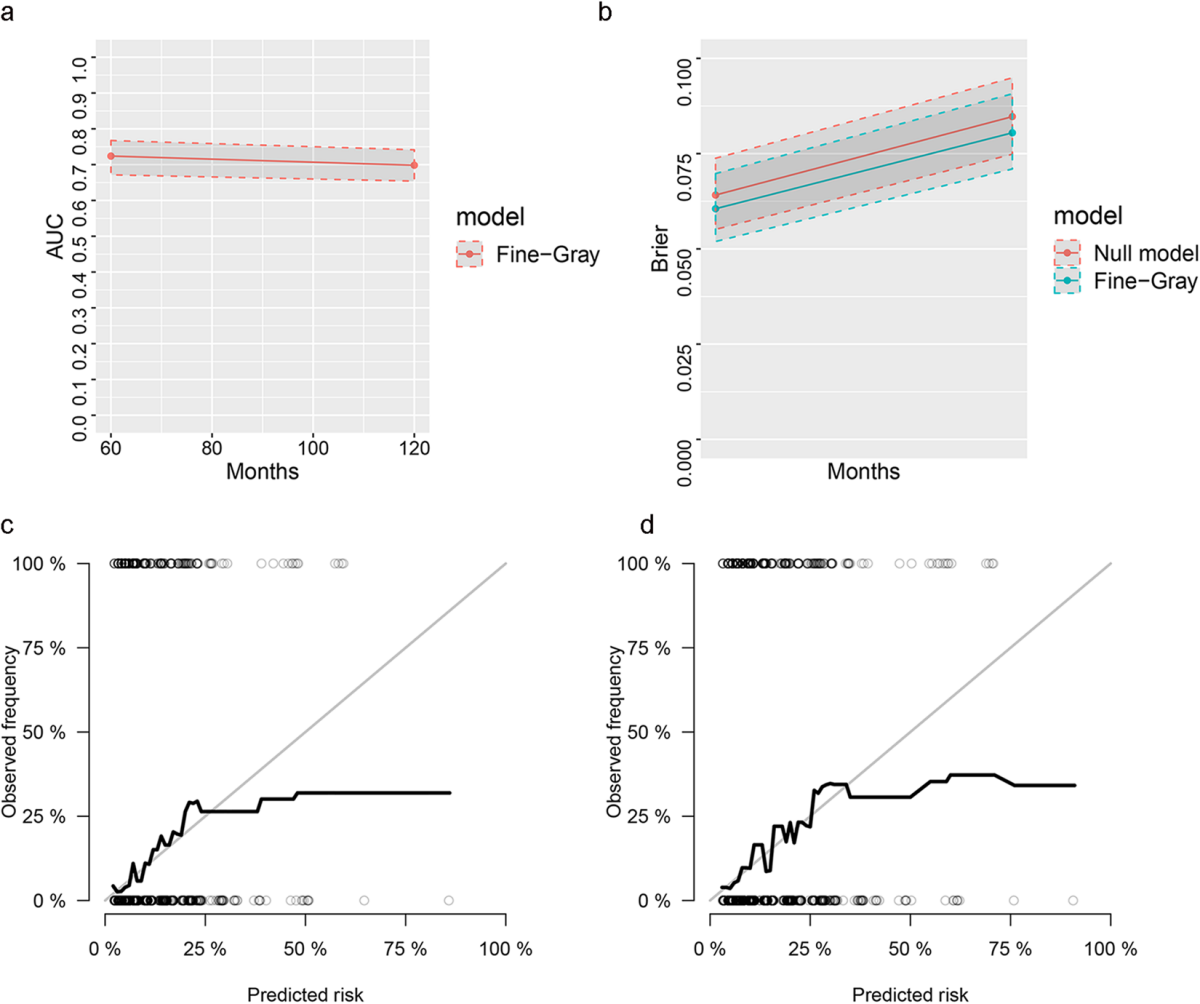
The discrimination and calibration of the nomogram of current study. (**a**) the time dependent AUC plot; (**b**) the time dependent brier score plot; c, the 5-year calibration plot; d, the 10-year calibration plot.

A penalized variable selection was performed for our SH model. As shown in Table 4, the LASSO, SCAD, and MCP analyses all identified tumor size, tumor stage, regional positive nodes number and marital status as the key variables in our SH model. The multivariate SH based nomogram was then constructed with these selected variables. A weighted total score calculated from each variable was used to estimate the 5-year and 10-year cause-specific death (Fig. 2). Time dependent the area under the curve of receiver operating characteristic curve (AUC, which was also referred as C-statistics) plots showed moderate discrimination of our nomogram (Fig. 3a). The AUC were 0.724 (95%confidence index (95%CI) = 0.671–0.767) and 0.698 (95%CI = 0.654–0.741) for 5-year and 10-year predictions, respectively. The brier score plot showed our nomogram model had lower score against the null model (Fig. 3b). The brier score was 0.0605 (95%CI = 0.0519–0.0698) and 0.0805 (95%CI = 0.0710–0.0907) for 5-year and 10-year predictions, respectively, indicating that our nomogram had good prediction. The calibration plots showed our nomogram predicted well in patients estimated at lower risk of deaths but not in patients estimated at high risk of deaths, which might come from that few patients were estimated with higher risk of death (Fig. 3c,d).

**Table 4:** Variable selection: Estimated coefficients (SEs) for the current SH model.

Characteristics	LASSO	SCAD	MCP
Age	0	0	0
Race	0	0	0
Laterality	0	0	0
Location	0	0	0
Grade	0	0	0
Tumor size	0.105	0.063	0.124
Tumor stage	0.442	0.621	0.558
Regional nodes positive	0.14	0.136	0.14
ER status	0	0	0
PR status	0	0	0
Marital status	0.197	0.195	0.296
Radiotherapy	0	0	0
Chemotherapy	0	0	0

LASSO, least absolute shrinkage and selection operator; SCAD, smoothly clipped absolute deviation (SCAD); MCP, measure–correlate-predict (MCP); ER, estrogen; PR, progesterone.

## Discussion

Most clinical cancer survival studies used Kaplan-Meier and Cox proportional hazards models to evaluate the prognostic value of their interests. Both models consider that there is a single cause to the event of interest, such as death. However, there were actually more causes (which are known as competing events) of deaths during the disease management, especially for cancers with long survival time such as breast cancer. Hence, the competing risk should be considered in the survival analysis. The current study was the first one to use competing risk models (CIF and SH models) to analyze the prognostic value of clinical variables to the BCSS of MCB and IDC. Our results showed IDC had a worse BCSS than MCB, which was consistent with previous studies^4,13–15^. Larger tumor size, a greater number of positive regional nodes and unmarried status were found to promote the progression of both MCB and IDC. We also found there were many discrepancies between IDC and MCB prognosis in variables included age, race, tumor location, tumor grade, tumor stage, ER and PR status, and records of radiotherapy or chemotherapy. This is currently the largest sample size study on MCB prognosis. Furthermore, we constructed the first competing model-based nomogram for estimating the 5-year and 10-year risk of death of MCB patients.

MCB patients were found to have younger age than IDC^3^. Our study with larger scale sample size confirmed this result. Younger patients exhibited a worse BCSS in IDC but there was no association between younger age and MCB BCSS.

Our results showed race played important parts in IDC. African American showed a relative worse outcome while patients from Asia exhibited better prognosis. However, there was limited influence of races in MCB. There was a study showed African American had a worse OS than Caucasians in MCB^10^. This study did not exclude severe patients that had no surgery chance. In the other hand, we only included early diagnosed patients who were resectable. We speculated African American might had more severe MCB patients which caused this difference.

Previous studies found MCB patients had higher grade, higher tumor stage and greater tumor size but meanwhile had favorable long-term distant relapse-free survival^2,3^. Our study showed similar results that MCB patients exhibited higher grade, higher tumor stage and larger tumor size than IDC. However, there was limited association of grade or tumor stage and MCB prognosis. Moreover, only tumor size over 30 millimetres had a significant worse outcome compared with tumors less than 10 millimetres in MCB. Increasing number of positive lymph nodes associated with worse OS and BCSS of early diagnosed breast cancer patients^16^. Our study also proved more positive lymph nodes would promote the cancer progression of both MCB and IDC patients.

Previous studies showed marriage was associated with the improvements in cardiovascular, endocrine, immune function, and cancer prognosis^17–19^. A study found that even after adjusting for known confounders, unmarried patients are at significantly higher risk of presentation with metastatic cancer, undertreatment, and death resulting from their cancer^17^. Having high levels of perceived social support, larger social network, and being married were found to be associated with decreases in relative risk for cancer mortality of 25%, 20%, and 12%, respectively^20^. We found the married status would improve the prognosis of MCB and IDC, which provide new evidences to the association between social support and cancer outcomes.

Positive ER or PR status were rarely found in MCB^9,10,15,21,22^. Our study found MCB had fewer ER or PR positive status than IDC. ER or PR positive status was often considered as good prognostic factors for breast cancer as the use of hormone treatment might help for certain patients^23^. Indeed, ER or PR positive status was found to be associated with better outcomes in IDC in our study. However, we found there was no association between ER or PR status and MCB prognosis. A previous study^24^ stratified breast cancer patients into 4 gene based clusters using hierarchical cluster analysis, among which Cluster B and Cluster A were both ER/PR positive and HER2 negative breast cancers. However, it found that Cluster B exhibited a worse cancer-specific survival than Cluster A. Moreover, previous study also found there were groups of ER positive breast cancers that were resistant to hormone study^25^. Furthermore, a study found no improvement of OS in MCB with adjuvant hormonal therapy^26^. The survival of ER/PR positive breast cancer might be influenced by other unknown biologic determinants. We speculated that ER/PR positive MCB might have cross gene expression profiling with these poor survival ER/PR positive breast cancer, which needed future genomic analysis to confirm.

Radiotherapy and chemotherapy were common adjuvant therapies for invasive breast cancers. However, it was often suggested that MCB had good prognosis and therefore may not benefit from systemic therapy. There was a study found chemotherapy would improve 5 and 10-year OS. However, the p value was 0.08, which might not be solid^26^. Previous study also showed radiotherapy had no association with MCB OS^10^. We found both radiotherapy and chemotherapy were not associated with MCB prognosis, which might be from the good prognostic feature of MCB. It is better to perform prognostic study among more severe MCB, such as patients with tumor size > 30 millimetres or had positive lymph nodes. In addition, according to NCCN^27^, the typical MCB is uncommon while many cases classified as MCB do not have all the pathologic features on subsequent pathologic review. High grade IDC patients might be mistakenly diagnosed as MCB. Therefore, the guideline recommends that cases diagnosed as MCB be treated as other IDC based on tumor size, grade and lymph node status. However, our study found the grade was limited associated with MCB prognosis, which might prove new hint to the concern of MCB treatments.

Nomogram could generate an individual probability of a clinical event by integrating various clinical variables, which is a valuable quantitative tool for personalized medicine^28^. Nomograms have been found to compare favorably to traditional TNM staging systems in many cancers^29,30^. To our best knowledge, our study constructed the first competing risk nomogram for MCB patients. The variables involved in current nomogram were easy to be obtained in clinical.

There were some limitations of current study. First, we only included patients with complete information of involved variables, which might cause selection bias. The excluding of patients with missing data would also reduce the sample size and statistical power of our study, especially for the patients with MCB, which is a rare histological type of breast cancer. Nevertheless, this is a study of MCB with the largest sample size. Second, despite the use of statistical matching, our study was based on retrospective cohort, which presented relative low level of clinical evidence. However, since the low incidence of MCB, it is hard to perform prospective study. Third, to obtain enough follow-up time, we excluded the data after 2010, which contained the information of HER2 status that was important for breast cancer prognosis. There was a study found hormone receptor positive/HER2 positive MCB had a better BCSS and OS than hormone receptor positive/HER2 negative MCB^7^. However, it needed to be further confirmed by larger simple scale study. Fourth, the population-based cancer registry (PBCR) data was limited by its absence of detailed information, and treatment information in PBCR data represent only the first course of treatment planned at diagnosis. For example, potential bias might exist in the data of radiotherapy and chemotherapy as many factors involved in determining the course of treatment will not be captured in the registry data. In addition, there was no information of hormone therapy in SEER, therefore the association between ER positive MCB and the resistant of hormone therapy needed further randomized controlled trials to confirm. Fifth, there was limited pathology information of MCB. Survival rates of atypical MCB was found to be worse than typical MCB^1,5,31^. The changes of diagnostic criteria in different time might cause bias^32^. Finally, to be note, the larger sample size could improve the statistical power. Although we involved with the largest sample size of MCB patients, it is much smaller than the IDC cohort. The differences between MCB and IDC might be caused by the difference of sample size. Therefore, the results of current study need to be further validated by study with larger sample size of MCB.

## Conclusion

In conclusion, we found MCB had a better BCSS than IDC. Larger tumor size, increasing number of positive lymph nodes and unmarried status were identified to promote the progression of MCB. ER or PR status and the use of radiotherapy or chemotherapy had no association with MCB prognosis. The competing risk nomogram of current study would be good clinical tool for prognostic estimation of MCB patients. Future larger sample studies are required to validate our findings.

## Methods

### Cohort selection

The cohort was obtained from 18 registries of SEER using SEER*Stat 8.3.5 software. As ER and PR status was registered since 1990 in SEER database, we involved patients diagnosed equal to or after 1990. To ensure adequate follow-up time, patients diagnosed after 2009 were excluded. Patients met the following inclusion criteria would be included: (1) it should be female primary MCB (8510/3: Medullary carcinoma, NOS) or IDC (ICD-O-3 Histology/behavior-8500/3) patients diagnosed between age 20 to79 who had surgery; (2) it should be unilateral invasive ductal carcinoma with location record; (3) it should include clinicopathological information for age at diagnosis, race, laterality, tumor location, grade, tumor size, information of Breast-Adjusted AJCC 6th Stage, number of positive regional nodes, ER and PR status, marital status, and radiotherapy or chemotherapy experience; (4) The survival time should over 3 months, and the vital status should be recoded for survival analyses; Any patient did not meet these criteria or lack of information for certain clinicopathological information would be excluded.

### Study variables

The following variables were extracted for the selected cohorts that included age at diagnosis, race (Caucasian, African American, American Indian/Alaska Native, Asian or Pacific Islander), laterality (right or left side), tumor location, grade (well-differentiated, moderately differentiated, poorly differentiated, undifferentiated or anaplastic), tumor size, information of Breast-Adjusted AJCC 6th Stage, number of positive regional nodes, ER and PR status (positive, borderline and negative), marital status, and radiotherapy and chemotherapy experience. The tumor location was defined through the SEER Site Specific Coding Modules (https://seer.cancer.gov/manuals/2016/appendixc.html), which comprised nipple, central portion, upper-inner quadrant, lower-inner quadrant, upper-outer quadrant, lower-outer quadrant, axillary tail, overlapping lesion and breast that is not otherwise specified. The Breast-Adjusted AJCC 6th Stage was roughly considered as I, II, III and IV. The widowed or single (never married or having a domestic partner) or divorced or separated patients was classified as unmarried. The value of age at diagnosis, tumor size and number of positive regional nodes were transformed into small categorical variables to fit the linear assumption. The median follow-up was estimated as the median observed survival time.

### Statistical analyses

The difference of each variable between MCB and IDC was analyzed by Chi-Squared tests. The CID were assessed for BCSM and other causes of death. Multivariate SH model was used to assess the BCSS. All the variables were involved in the multivariate SH analysis. HR and 95%CI were calculated. Statistical matching of all variables of MCB and IDC was performed by CEM, which was identified to have the ability to achieve lower levels of imbalance, model dependence, and bias than Propensity Score matching^33,34^. The standardized difference in means of all variables before and after CEM was calculated. Histograms of the distance measure before and after matching were plotted to estimate the efficacy of CEM. There were 4 histograms provided: the original treated and control groups and the matched treated and control groups. The increase of similarity of matched treated and control groups were considered the success of statistical matching^35^. The matched data was then analyzed by CIF test and multivariate SH model.

Multivariate SH model-based nomogram was constructed from the multivariate logistic regression model to predict the 5-year and 10-year cause-specific death of MCB patients. Over-fitting can become a serious problem when there are many potential variables in one predict model^36^. The variable selection was required to improve the prediction, and interpretation of our nomogram. Therefore, penalized variable selection was performed for our SH model by using techniques of least absolute shrinkage and selection operator (LASSO), smoothly clipped absolute deviation (SCAD) and measure–correlate-predict (MCP). The selected variables would be involved in the SH model based nomogram. Internal validation of nomogram was estimated by discrimination and calibration. Discrimination is the ability of a model to distinguish between patients who have an event from those who do not. Calibration assesses how far the predictions are from the actual outcomes^28^. AUC was evaluated for the discrimination of nomograms. The AUC ranges from 0.5–1.0, with 0.5 indicates the outcomes is completely random and 1.0 indicates the perfect discrimination. Calibration curve was used to assess the calibration by comparing how close the nomogram estimated risk line was to the observed risk line in an axis. The brier score for an event at a time is defined as the expected squared distance between the observed status at that time and the predicted probability. Hence, a smaller value of brier score suggests a better model^37^. The brier score could account the discrimination and calibration at the same time^38^. These validation methods of nomogram were performed with 1,000 bootstraps to avoid the bias from overfitting. The bootstraps methodology is commonly used in the internal validation of nomograms whereby the model is iteratively applied to randomly selected sample sets of the original cohort^29^.

All the statistical analyses were performed using R version 3.4.2. R package “cmprsk” was used to perform the CIF test and multivariate SH analysis. CEM analysis was performed by R package “MatchIt”. The variable selection of SH model was performed by R package “crrp”. The competing risk nomogram was plotted by R packages “mstate” and “regplot”. The AUC and brier score were calculated by R package “riskRegression” and plotted by “ggplot2”. The calibration curve was drawn by R package “riskRegression”. A p-value less than 0.05 was considered statistically significant.

## Supplementary information

Supplementary Information

Supplementary Information 2

Supplementary Information 3
