# Supplementary material for: Competing Risk Analyses of Medullary Carcinoma of Breast in Comparison to Infiltrating Ductal Carcinoma
Source: Sci Rep. 2020 Jan 17;10:560. doi: 10.1038/s41598-019-57168-2 (PMC6969020; doi:10.1038/s41598-019-57168-2)
Supplement: Supplementary file 1 — Supplementary Information [file 41598_2019_57168_MOESM1_ESM.docx]

**Competing Risk Analyses of Medullary Carcinoma of Breast in Comparison to Infiltrating Ductal Carcinoma**

Dongjun Dai^1^, Rongkai Shi^1^, Zhuo Wang^1^, Yiming Zhong^1^, Vivian Y Shin^3^, Hongchuan Jin^2^, Xian Wang^1,*^

**Supplementary Table S1.** The proportion of deaths due to cancer and other causes in each variable

| Characteristics | MCB | | | |  | IDC | | | |
| --- | --- | --- | --- | --- | --- | --- | --- | --- | --- |
|  | BCSM | | Deaths of other cause | |  | BCSM | | Deaths of other cause | |
|  | No. of patients | % | No. of patients | % |  | No. of patients | % | No. of patients | % |
| **Age** |  |  |  |  |  |  |  |  |  |
| 20-29 | 7 | 10.61% | 3 | 4.55% |  | 514 | 25.10% | 79 | 3.86% |
| 30-39 | 59 | 11.28% | 32 | 6.12% |  | 4385 | 21.01% | 753 | 3.61% |
| 40-49 | 113 | 10.20% | 74 | 6.68% |  | 9569 | 13.84% | 3215 | 4.65% |
| 50-59 | 100 | 10.48% | 103 | 10.80% |  | 10849 | 12.37% | 7290 | 8.31% |
| 60-69 | 76 | 12.08% | 159 | 25.28% |  | 9178 | 11.58% | 15177 | 19.14% |
| 70-79 | 41 | 13.67% | 137 | 45.67% |  | 7513 | 12.41% | 23893 | 39.46% |
| **Race** |  |  |  |  |  |  |  |  |  |
| Caucasian | 262 | 10.26% | 386 | 15.11% |  | 32783 | 12.47% | 42970 | 16.35% |
| African American | 107 | 14.25% | 94 | 12.52% |  | 6029 | 20.38% | 4544 | 15.36% |
| American Indian/Alaska Native | 5 | 16.67% | 3 | 10.00% |  | 279 | 17.60% | 251 | 15.84% |
| Asian or Pacific Islander | 22 | 8.98% | 25 | 10.20% |  | 2917 | 11.40% | 2642 | 10.32% |
| **Laterality** |  |  |  |  |  |  |  |  |  |
| Right - origin of primary | 200 | 11.14% | 270 | 15.04% |  | 20407 | 12.95% | 24713 | 15.68% |
| Left - origin of primary | 196 | 10.98% | 238 | 13.33% |  | 21601 | 13.34% | 25694 | 15.87% |
| **Location** |  |  |  |  |  |  |  |  |  |
| Nipple | 2 | 33.33% | 2 | 33.33% |  | 229 | 15.87% | 340 | 23.56% |
| Central portion of breast | 15 | 13.76% | 17 | 15.60% |  | 2516 | 14.61% | 3196 | 18.56% |
| Upper-inner quadrant of breast | 48 | 11.76% | 50 | 12.25% |  | 4294 | 11.81% | 5118 | 14.08% |
| Lower-inner quadrant of breast | 21 | 9.55% | 22 | 10.00% |  | 2412 | 12.83% | 3065 | 16.31% |
| Upper-outer quadrant of breast | 153 | 10.49% | 226 | 15.49% |  | 14626 | 12.38% | 18847 | 15.96% |
| Lower-outer quadrant of breast | 29 | 10.32% | 43 | 15.30% |  | 2969 | 13.13% | 3378 | 14.94% |
| Axillary tail of breast | 5 | 8.33% | 10 | 16.67% |  | 333 | 14.58% | 327 | 14.32% |
| Overlapping lesion of breast | 79 | 11.11% | 96 | 13.50% |  | 8719 | 13.08% | 10336 | 15.50% |
| Breast, NOS | 44 | 13.50% | 42 | 12.88% |  | 5910 | 16.37% | 5800 | 16.07% |
| **Grade** |  |  |  |  |  |  |  |  |  |
| Well differentiated; Grade I | 4 | 16.67% | 4 | 16.67% |  | 1874 | 3.58% | 8164 | 15.59% |
| Moderately differentiated; Grade II | 30 | 16.48% | 26 | 14.29% |  | 12540 | 10.02% | 20647 | 16.50% |
| Poorly differentiated; Grade III | 219 | 10.64% | 232 | 11.27% |  | 24001 | 19.63% | 16717 | 13.67% |
| Undifferentiated; anaplastic; Grade IV | 19 | 8.37% | 44 | 19.38% |  | 1180 | 22.40% | 905 | 17.18% |
| Unknown | 124 | 11.39% | 202 | 18.55% |  | 2413 | 16.65% | 3974 | 27.42% |
| **Tumor size** |  |  |  |  |  |  |  |  |  |
| <=1cm | 19 | 6.01% | 45 | 14.24% |  | 3421 | 4.34% | 12590 | 15.97% |
| <=2cm | 125 | 8.74% | 207 | 14.47% |  | 13228 | 10.23% | 21277 | 16.46% |
| <=3cm | 129 | 11.27% | 149 | 13.01% |  | 11757 | 18.60% | 9777 | 15.47% |
| <=4cm | 58 | 14.01% | 64 | 15.46% |  | 5888 | 25.29% | 3512 | 15.08% |
| <=5cm | 31 | 20.53% | 29 | 19.21% |  | 3156 | 29.95% | 1490 | 14.14% |
| >5cm | 34 | 27.64% | 14 | 11.38% |  | 4558 | 31.61% | 1761 | 12.21% |
| **Tumor stage** |  |  |  |  |  |  |  |  |  |
| I | 80 | 6.01% | 183 | 13.74% |  | 8355 | 5.27% | 26419 | 16.66% |
| II | 226 | 11.52% | 287 | 14.64% |  | 17194 | 14.64% | 17693 | 15.06% |
| III | 86 | 31.27% | 35 | 12.73% |  | 14140 | 35.42% | 5837 | 14.62% |
| IV | 4 | 33.33% | 3 | 25.00% |  | 2319 | 64.61% | 458 | 12.76% |
| **Regional nodes positive** |  |  |  |  |  |  |  |  |  |
| >=10 | 24 | 43.64% | 7 | 12.73% |  | 5611 | 50.12% | 1591 | 14.21% |
| 0 | 196 | 7.61% | 362 | 14.06% |  | 15325 | 7.34% | 34407 | 16.49% |
| 1-3 | 122 | 15.60% | 115 | 14.71% |  | 12791 | 17.10% | 10723 | 14.33% |
| 4-9 | 54 | 31.95% | 24 | 14.20% |  | 8281 | 33.32% | 3686 | 14.83% |
| **ER status** |  |  |  |  |  |  |  |  |  |
| Negative | 306 | 10.89% | 355 | 12.63% |  | 16052 | 20.26% | 10744 | 13.56% |
| Positive | 87 | 11.97% | 140 | 19.26% |  | 25664 | 10.74% | 39387 | 16.48% |
| Borderline | 3 | 6.98% | 13 | 30.23% |  | 292 | 21.44% | 276 | 20.26% |
| **PR status** |  |  |  |  |  |  |  |  |  |
| Negative | 321 | 10.97% | 395 | 13.50% |  | 20161 | 18.34% | 16302 | 14.83% |
| Positive | 71 | 11.43% | 105 | 16.91% |  | 21436 | 10.35% | 33621 | 16.23% |
| Borderline | 4 | 11.76% | 8 | 23.53% |  | 411 | 16.61% | 484 | 19.56% |
| **Marital status** |  |  |  |  |  |  |  |  |  |
| Married | 214 | 9.40% | 264 | 11.60% |  | 24691 | 12.30% | 25813 | 12.86% |
| Unmarried | 182 | 13.96% | 244 | 18.71% |  | 17317 | 14.58% | 24594 | 20.70% |
| **Radiotherapy** |  |  |  |  |  |  |  |  |  |
| No | 179 | 11.25% | 274 | 17.22% |  | 20741 | 14.62% | 27523 | 19.40% |
| Yes | 217 | 10.91% | 234 | 11.76% |  | 21267 | 11.97% | 22884 | 12.88% |
| **Chemotherapy** |  |  |  |  |  |  |  |  |  |
| No | 156 | 11.17% | 292 | 20.90% |  | 15723 | 9.14% | 37194 | 21.62% |
| Yes | 240 | 10.99% | 216 | 9.89% |  | 26285 | 17.82% | 13213 | 8.96% |

MCB, medullary carcinoma of the breast; IDC, invasive ductal carcinoma of breast; BCSM, breast cancer specific mortality; ER, estrogen; PR, progesterone.
