# Supplementary material for: Competing Risk Analyses of Medullary Carcinoma of Breast in Comparison to Infiltrating Ductal Carcinoma
Source: Sci Rep. 2020 Jan 17;10:560. doi: 10.1038/s41598-019-57168-2 (PMC6969020; doi:10.1038/s41598-019-57168-2)
Supplement: Supplementary file 2 — Supplementary Information 2 [file 41598_2019_57168_MOESM2_ESM.docx]

**Competing Risk Analyses of Medullary Carcinoma of Breast in Comparison to Infiltrating Ductal Carcinoma**

Dongjun Dai^1^, Rongkai Shi^1^, Zhuo Wang^1^, Yiming Zhong^1^, Vivian Y Shin^3^, Hongchuan Jin^2^, Xian Wang^1,*^

**Supplementary Fig. S1.** Standardized difference in means of variables between MCB and IDC before and after CEM


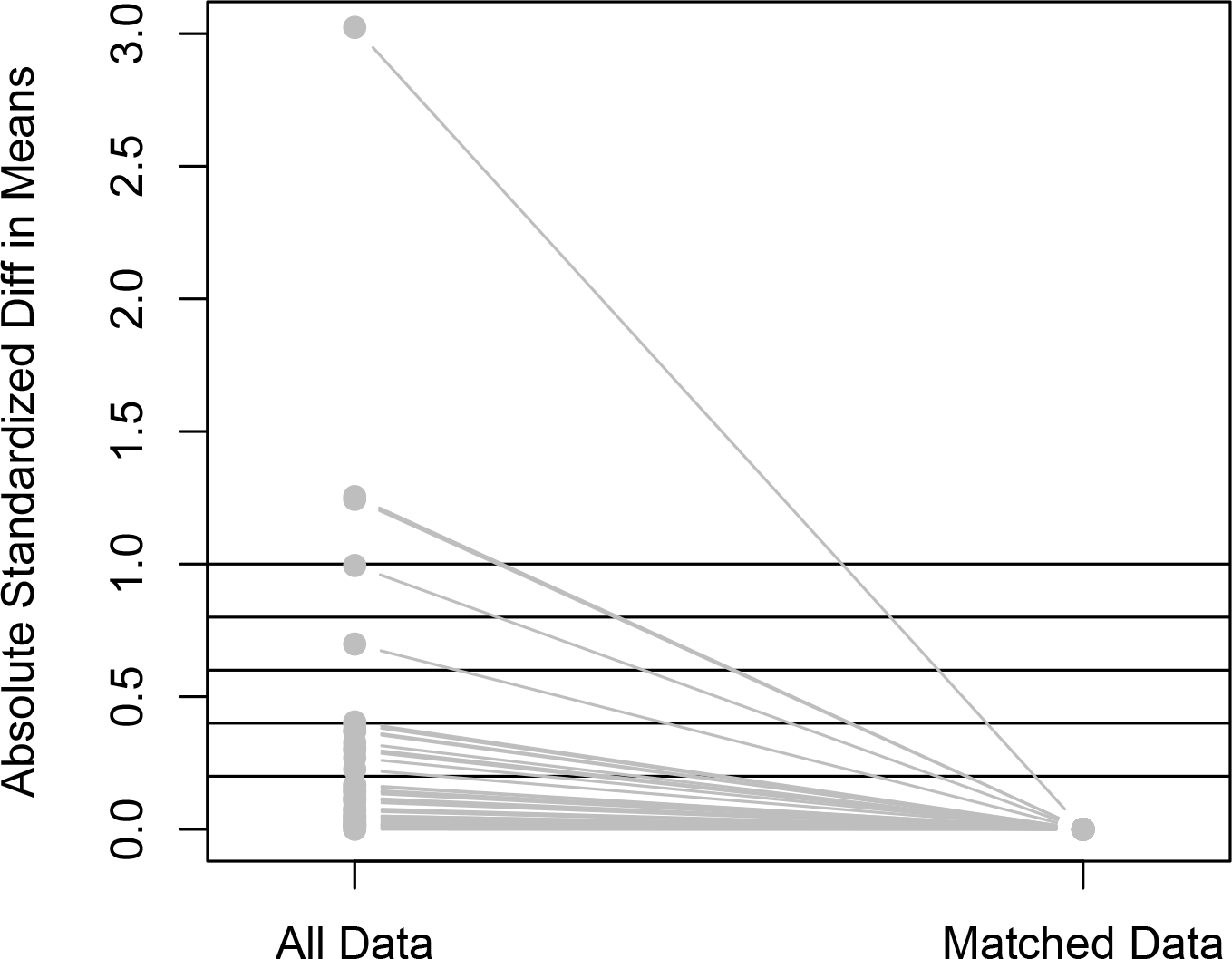


**Legends:** The standard mean difference between MCB and IDC of all variables was eliminated after CEM, a circle represents one variable.
